# The human brain in the transhumanist mindset. A neuroethical critique of the neuroscience paradigm

**DOI:** 10.1192/j.eurpsy.2022.1723

**Published:** 2022-09-01

**Authors:** J. Galvañ

**Affiliations:** Hospital General Universitario Gregorio Marañón, Psychiatry, Madrid, Spain

**Keywords:** Bioethical, Neuroscience, Phylosophy, mind

## Abstract

**Introduction:**

Neuroscience advances open the debate on improving brain functionality and human behavior. Transhumanism advocates the use of biotechnology for the betterment of man, transcending into another nature. Neuroethics marks limits of the application and experimentation in neuroscience, also proposing an interdisciplinary philosophical reflection valuing the multidimensionality of human mind.

**Objectives:**

To analyze the transhumanist approach of domining human nature controlling cognitive and moral functions through technique. A critique from neuroethics in an interdisciplinary key to evaluate the complexity of mental functionality and the derived issues that go beyond the scientific scope, with the help of philosophy.

**Methods:**

A bibliographic review on neuroscience and neuroethics is carried out, finding a core consensus in the warning of the biopsychosocial complexity of the set of realities that shape the human being, facing a reductionist vision of neuroscience.

**Results:**

Despite the advances in biotechnology and neuroscientific research, the transhumanist approach of brain enhancement transgressing human reality and reducing its nature to a mechanical question that can be controlled through psychopharmacological resources, becomes dystopian due to the lack of ontological continuity in the deconstruction of the human being in a set of neural circuits, and the lack of consideration of all the dimensions that configure the human mind and existence.

**Conclusions:**

An interdisciplinary vision is necessary to analyze the human mind, avoiding falling into the brain reductionism of the neuroscientific paradigm, for an holistic understanding of the human mind and behaviors, beside the integration of a philosophical reflection to join neurobiology and moral dimensions, in a humanist return from transhumanism.

**Disclosure:**

No significant relationships.

